# Targeting ACLY sensitizes castration-resistant prostate cancer cells to AR antagonism by impinging on an ACLY-AMPK-AR feedback mechanism

**DOI:** 10.18632/oncotarget.9666

**Published:** 2016-05-27

**Authors:** Supriya Shah, Whitney J. Carriveau, Jinyang Li, Sydney L. Campbell, Piotr K. Kopinski, Hee-Woong Lim, Natalie Daurio, Sophie Trefely, Kyoung-Jae Won, Douglas C. Wallace, Constantinos Koumenis, Anthony Mancuso, Kathryn E. Wellen

**Affiliations:** ^1^ Department of Cancer Biology, University of Pennsylvania Perelman School of Medicine, Philadelphia, PA 19104, USA; ^2^ Department of Genetics, University of Pennsylvania Perelman School of Medicine, Philadelphia, PA 19104, USA; ^3^ Department of Radiation Oncology, University of Pennsylvania Perelman School of Medicine, Philadelphia, PA 19104, USA; ^4^ Department of Radiology, University of Pennsylvania Perelman School of Medicine, Philadelphia, PA 19104, USA; ^5^ Center for Mitochondrial and Epigenomic Medicine, Children's Hospital of Philadelphia, Philadelphia, PA 19104, USA; ^6^ Howard Hughes Medical Institute, Philadelphia, PA 19104, USA

**Keywords:** acetyl-CoA, prostate cancer, fatty acid metabolism, AMPK, ER stress

## Abstract

The androgen receptor (AR) plays a central role in prostate tumor growth. Inappropriate reactivation of the AR after androgen deprivation therapy promotes development of incurable castration-resistant prostate cancer (CRPC). In this study, we provide evidence that metabolic features of prostate cancer cells can be exploited to sensitize CRPC cells to AR antagonism. We identify a feedback loop between ATP-citrate lyase (ACLY)-dependent fatty acid synthesis, AMPK, and the AR in prostate cancer cells that could contribute to therapeutic resistance by maintaining AR levels. When combined with an AR antagonist, ACLY inhibition in CRPC cells promotes energetic stress and AMPK activation, resulting in further suppression of AR levels and target gene expression, inhibition of proliferation, and apoptosis. Supplying exogenous fatty acids can restore energetic homeostasis; however, this rescue does not occur through increased β-oxidation to support mitochondrial ATP production. Instead, concurrent inhibition of ACLY and AR may drive excess ATP consumption as cells attempt to cope with endoplasmic reticulum (ER) stress, which is prevented by fatty acid supplementation. Thus, fatty acid metabolism plays a key role in coordinating ER and energetic homeostasis in CRPC cells, thereby sustaining AR action and promoting proliferation. Consistent with a role for fatty acid metabolism in sustaining AR levels in prostate cancer *in vivo*, *AR* mRNA levels in human prostate tumors correlate positively with expression of *ACLY* and other fatty acid synthesis genes. The ACLY-AMPK-AR network can be exploited to sensitize CRPC cells to AR antagonism, suggesting novel therapeutic opportunities for prostate cancer.

## INTRODUCTION

Prostate cancer is responsible for nearly 30,000 deaths each year in the United States alone [1]. While localized prostate cancer has a favorable prognosis, some patients present with or develop metastatic disease. The androgen receptor (AR) plays a central role in prostate tumorigenesis at both early and late stages of the disease. Patients with metastatic prostate cancer typically respond well to androgen deprivation therapy. However, tumors eventually develop resistance mechanisms that allow them to grow at castrate levels of androgen, resulting in a terminal disease known as castration-resistant prostate cancer (CRPC). In CRPC, AR activity frequently remains crucial for disease progression, with tumors acquiring resistance mechanisms that enable AR transcriptional activity even with very low circulating levels of its canonical ligand [2–7]. Second-generation AR antagonists such as enzalutamide (ENZ), which inhibit ligand binding to the AR, extend the lives of some CRPC patients, but are not curative [4, 8, 9]. Therefore, identification of more effective therapeutic approaches is urgently needed for CRPC patients.

In addition to aberrant AR signaling, prostate cancer cells exhibit metabolic rewiring, including upregulation of fatty acid synthesis [10, 11]. Acetyl-CoA, the essential building block for fatty acid metabolism, plays crucial roles at the interface of metabolism, signaling, and gene regulation [12, 13]. It is produced within mitochondria from pyruvate, as well as from catabolism of fatty acids and amino acids, in order to sustain the TCA cycle and mitochondrial ATP production. Citrate, the condensation product of acetyl-CoA and oxaloacetate, can be exported from mitochondria and cleaved by ATP-citrate lyase (ACLY), generating acetyl-CoA within the cytoplasm and nucleus. Outside of mitochondria, acetyl-CoA is used for *de novo* synthesis of fatty acids and cholesterol, as well as for the acetylation of histones and numerous other proteins [12–15].

The PI3K-AKT-mTORC1 pathway is commonly activated in prostate cancer, most frequently via *PTEN* loss; in metastatic disease, 49% of tumors exhibit alterations in this pathway [16]. Constitutive PI3K-AKT pathway activation in cancer cells is associated with elevated ACLY-dependent acetyl-CoA production and histone acetylation [17]. Moreover, the PI3K-AKT-mTORC1 pathway also drives *de novo* fatty acid synthesis [18–22].

There is substantial prior evidence that fatty acid metabolism is upregulated in prostate cancer and is regulated by the AR [10, 11, 23–25]. For example, in a prostate cancer xenograft model, fatty acid synthesis genes are highly expressed in the primary tumor, suppressed following castration, and then upregulated as resistant tumors emerge, suggesting potential involvement of fatty acid metabolism in CRPC development [23]. Inhibiting lipogenic enzymes such as fatty acid synthase (FASN), acetyl-CoA carboxylase (ACC), or ACLY produces anti-cancer effects both in prostate cancer cell lines and mouse models [10, 26–30]. Activation of AMP-activated protein kinase (AMPK), which inhibits fatty acid synthesis by phosphorylating ACC [31], also inhibits prostate tumorigenesis [10, 32–35]. Acetyl-CoA metabolism may also promote growth and proliferation through gene regulation. In yeast, high acetyl-CoA levels induce histone acetylation at the promoters of genes involved in growth and proliferation [36, 37]. Similarly, acetyl-CoA promotes global histone acetylation and expression of pro-proliferative gene expression in cancer cells [15, 17, 38], although the underlying mechanisms of gene regulation by acetyl-CoA are not fully clear. Histone acetylation has been shown to be important for AR recruitment to chromatin and transcriptional activity [39]. Thus, elevated production of nuclear-cytoplasmic acetyl-CoA may support prostate tumor growth through both lipid metabolism and gene regulation.

In this study, we identify a signaling network between ACLY-dependent fatty acid metabolism, AMPK, and the AR. We show that ACLY inhibition potentiates the action of ENZ in suppressing AR function, and combining the two drugs strongly inhibits proliferation and induces apoptosis in CRPC cells. Prior studies have demonstrated that the PI3K-AKT and AR pathways inhibit one another, and that AR inhibition drives hyper-activation of AKT [40, 41]. Constitutive AKT-mTORC1 pathway activation has also been shown to exacerbate dependence on *de novo* fatty acid synthesis for endoplasmic reticulum (ER) homeostasis [42–44]. In this study, we show that simultaneous targeting of ACLY and AR exploits these interconnected signaling and metabolic pathways to induce ER stress and AMPK activation, which then drives further AR suppression and cell death. Energetic stress due to concurrent inhibition of ACLY and AR appears to result at least in part from excess ATP consumption as cells attempt to resolve ER stress. Thus, these data identify a novel mechanism linking cellular metabolism to transcriptional control in prostate cancer cells and point towards new opportunities to exploit the ACLY-AMPK-AR network for therapeutic benefit in patients with CRPC, a disease for which to date there remains no cure.

## RESULTS

### ACLY inhibition sensitizes CRPC cells to AR antagonism

**Figure 1:**
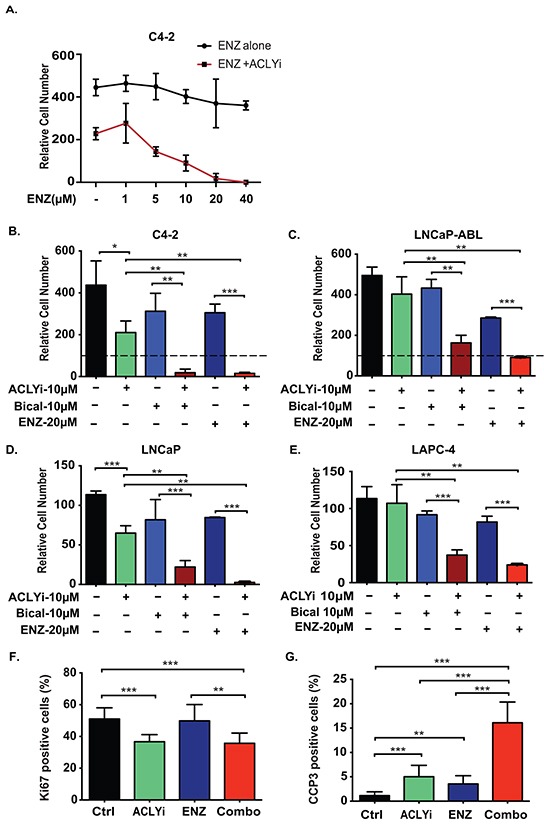
ACLY inhibition sensitizes castration-resistant prostate cancer cells to AR antagonism under androgen depletion **A.** C4-2 cells were cultured in androgen-depleted conditions for 24 hours and then treated with doses of enzalutamide (ENZ) ranging from 0-40 μM, in the presence or absence of 10 μM ACLY inhibitor (ACLYi; BMS-303141), for 72 hours. Cell number was quantitated relative to cell number at the start of inhibitor treatment and initial cell number set at 100. **B-E.** Indicated cell lines were cultured for 72 hours in androgen-depleted conditions, +/− ACLYi, ENZ, or Bicalutamide (Bical). Cell number was quantitated relative to starting cell number, set at 100. **F, G.** Cells were treated for 24 hours with indicated inhibitors in androgen-depleted conditions. Immunofluorescent imaging was used to detect the proliferation indicator Ki67 (F) or apoptotic indicator cleaved caspase 3 (CCP3) (G). Data was quantified and graphed from 5 fields. For all values, mean +/− SEM of triplicates is graphed; *, p<0.05; **, p<0.01, ***, p<0.001.

CRPC cells depend on the AR, which remains transcriptionally active in CRPC even in the absence of exogenous ligand [5–7]. As a result, C4-2 CRPC cells continued to proliferate in androgen-depleted conditions and were only minimally inhibited by enzalutamide (ENZ) (Figure 1A). *ACLY* expression is elevated in human prostate cancer and correlates with *AR* expression (Supplementary Figure S1A, S1B). An ACLY inhibitor (ACLYi; BMS-303141) impaired proliferation or induced death in androgen-depleted CRPC cells in a dose-dependent manner (Supplementary Figure S1C). At a dose of ACLYi that moderately impaired proliferation, cells were strongly sensitized to ENZ (Figure 1A). Combining ACLYi with either of two AR antagonists (ENZ or bicalutamide) potently suppressed growth in both CRPC and hormone naïve AR+ prostate cancer cell lines (Figure 1B-1E). In contrast, cells that do not rely on the AR for proliferation such as PC-3 prostate cancer cells and 3T3-L1 preadipocytes were minimally impacted by the combination of ACLYi and ENZ (denoted hereafter as Combo) (Supplementary Figure S1D, S1E). The anti-proliferative effect in C4-2 cells, assessed by Ki67 positivity, was predominantly mediated by ACLYi, while the pro-apoptotic effect, assessed by cleaved-caspase 3, was strongly potentiated by the Combo (Figure 1F, 1G). Similar results were obtained in C4-2 cells when ENZ was combined with either shRNA-mediated silencing of ACLY or a structurally distinct ACLY inhibitor, SB204990 [45] (Supplementary Figure S1F, S1G). When cells were cultured in standard FBS (not hormone-depleted), significant growth suppression was also observed with each inhibitor on its own, and this suppression was enhanced by combining the two (Supplementary Figure S1H). Thus, co-targeting ACLY and the AR effectively suppresses growth both in the presence and absence of exogenous androgen.

### Combining ACLY and AR inhibitors suppresses AR target gene expression

**Figure 2:**
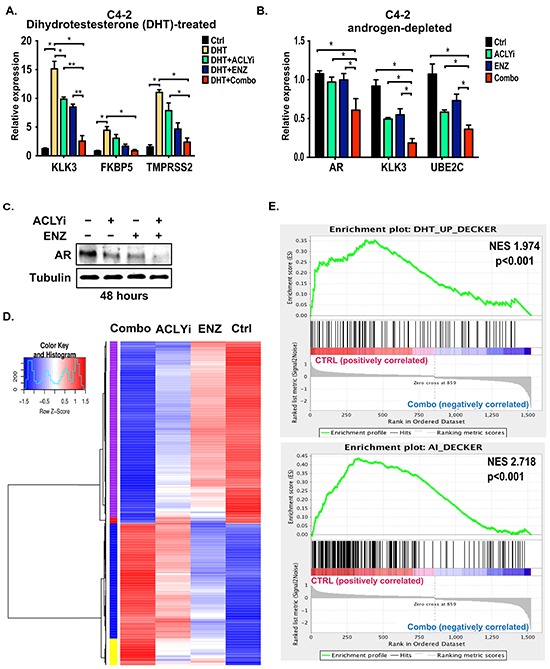
Combining ACLY and AR inhibitors suppresses AR target gene expression **A.** C4-2 cells were cultured in androgen-depleted conditions for 24 hours and then treated with DHT (10 nM), +/− ACLYi (10 μM), +/− ENZ (20 μM) for an additional 24 hours. Gene expression was analyzed by Q-RT-PCR and normalized to 18S rRNA. Combo denotes ACLYi + ENZ. **B.** C4-2 cells were cultured in androgen-depleted conditions for 24 hours and then treated +/− ACLYi (10 μM), +/− ENZ (20 μM) for an additional 24 hours. Gene expression was analyzed by Q-RT-PCR and normalized to B2M. **C.** Cells were treated with ACLYi or ENZ for 48 hours in androgen-depleted conditions and AR levels analyzed by Western blot. **D.** C4-2 cells in androgen-depleted conditions were treated with vehicle, ACLYi alone, ENZ alone, or the inhibitor Combo. Genes differentially expressed in any two comparisons (FDR<0.0001) were clustered and are represented in the heat map. See Supplementary Table S1 for associated gene list and cluster ID. **E.** Gene set enrichment analysis (GSEA) was used to test whether the “DHT-Up” and “Androgen Independent” gene signatures, as defined by Decker et al [7], were enriched in Ctrl vs. Combo-treated cells. Both of these gene sets were enriched in control C4-2 cells as compared to Combo-treated C4-2 cells. Normalized enrichment score (NES) and p-values are indicated. See Supplementary Figure S2 for comparisons between Ctrl vs. ACLYi alone. For all bar graphs, mean +/− SEM of triplicates is graphed; *, p<0.05; **, p<0.01

Ligand-dependent and ligand-independent binding of the AR to chromatin, as well as androgen-dependent and androgen-independent CRPC gene signatures, have been previously defined in C4-2 cells [7]. “Androgen-independent” genes are those whose expression is enriched in C4-2 cells compared to the parental hormone-dependent cell line LNCaP. The AR also binds many of these genes in a ligand-independent manner [7]. As noted above, these cells continue to proliferate in the absence of androgen; however, both the AR and “androgen-independent” genes remain crucial for their growth [7]. We used these cells as a model of CRPC to interrogate the effects of ACLY/AR inhibition on gene expression in CRPC cells. C4-2 cells were treated with DHT in the presence or absence of ENZ and/or ACLYi. As expected, AR target genes, including *KLK3*, *FKBP5*, and *TMPRSS2*, were induced by DHT, and their induction was inhibited by ENZ, which inhibits ligand binding to the AR [9] (Figure 2A). ACLYi also significantly suppressed expression of *KLK3* [encoding prostate specific antigen, (PSA)] on its own, and further suppressed its expression in the presence of ENZ (Figure 2A). Under androgen-depleted conditions, the Combo suppressed expression of the *AR* itself, as well as *KLK3* and *UBE2C*, an androgen-independent AR target [6] (Figure 2B). Similar effects of the inhibitors on AR target gene expression in both the presence and absence of DHT were also observed in LNCaP-Abl CRPC cells (Supplementary Figure S2A). Notably, AR protein levels were also markedly reduced in androgen-depleted ACLYi-treated cells, an effect that was magnified by the Combo (Figure 2C). ACLY silencing combined with an AR antagonist similarly suppressed AR levels (Supplementary Figure S4B).

To comprehensively evaluate the impact of the Combo on gene expression, we conducted RNA-sequencing on C4-2 cells treated for 24 hours with ACLYi, ENZ, or the Combo under hormone-depleted conditions. Four major gene clusters were identified- 1) those suppressed by ACLYi and further suppressed by Combo (purple), 2) those suppressed by ENZ and further suppressed by Combo (red), 3) those induced by ACLYi and further enhanced by Combo (blue), and 4) those induced by both ACLYi and ENZ individually and further induced by Combo (yellow) (Figure 2D, Supplementary Table S1). Using Gene Set Enrichment Analysis (GSEA), we found that ACLYi suppressed expression of previously defined “acetyl-CoA-upregulated” genes and enriched “acetyl-CoA-downregulated” genes [17] (Supplementary Figure S2B). ACLYi alone also significantly suppressed “androgen-independent” genes (Supplementary Figure S2C), and combining ACLYi and ENZ further accentuated these effects, resulting in potent suppression of both “DHT-induced” and “androgen-independent” gene signatures [7] (Figure 2E).

### Combining ACLY and AR inhibitors induces ER stress

**Figure 3:**
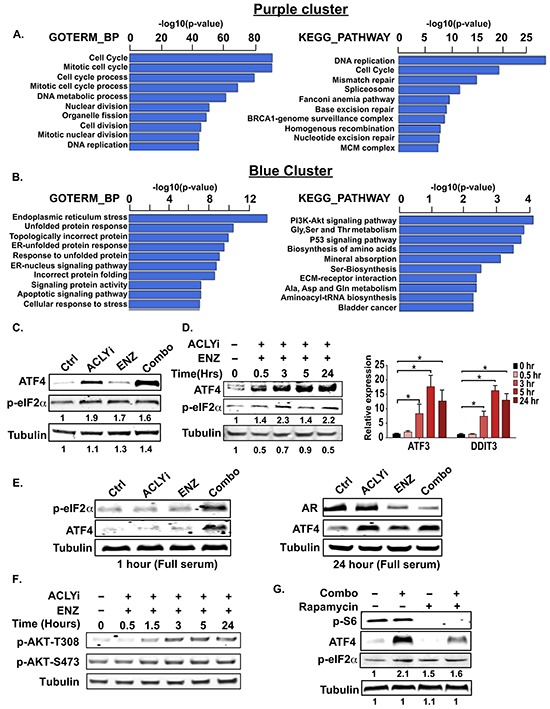
Combining ACLY and AR inhibitors induces endoplasmic reticulum stress **A, B.** RNA-sequencing data was analyzed using the HOMER tool [46]. GOTERM_BP and KEGG_PATHWAY analyses are shown for the purple cluster (downregulated genes, A) and blue cluster (upregulated genes, B). **C.** C4-2 cells were treated with indicated inhibitors for 24 hours under androgen deprivation and analyzed by Western blot. **D.** C4-2 cells were treated with ACLYi + ENZ under androgen deprivation for indicated times and analyzed by Western blot and Q-RT-PCR. **E.** C4-2 cells were treated for 1 or 24 hours with indicated inhibitors in standard serum (not androgen depleted) and analyzed by Western blot. **F.** AKT phosphorylation was analyzed by Western blot after treating C4-2 cells as indicated under androgen deprivation. **G.** C4-2 cells were pre-treated for 1 hour with rapamycin, then treated with Combo for an additional hour and analyzed by Western blot. For all bar graphs, mean +/− SEM of triplicates is graphed; *, p<0.05

To gain further insight into additional pathways that are regulated by the inhibitor Combo, we employed gene ontology analysis using the HOMER tool [46], focusing on the two largest clusters. Within the purple cluster, genes involved in cell cycle and DNA replication and repair predominated (Figure 3A, Supplementary Figure S3A), consistent with the observed reduction in proliferation with ACLYi or Combo. The blue cluster was strikingly defined by an endoplasmic reticulum (ER) stress signature (Figure 3B, Supplementary Figure S3B).

The unfolded protein response (UPR) is controlled by three sensors within the ER membrane: ATF6, IRE1, and PERK. Disturbances in lipid homeostasis or accumulation of unfolded proteins are detected within the ER lumen and mediate adaptive signaling and transcriptional responses to restore ER homeostasis. The UPR can also exert pro-apoptotic functions when ER stress is prolonged or unresolvable [47]. Notably, the AR differentially regulates UPR pathways, promoting the IRE1 arm while suppressing the PERK arm and its downstream pro-apoptotic outputs such as CHOP [48]. Among ER-stress signature genes strongly induced by the inhibitor Combo were *ATF3*, *DDIT3* (CHOP) and CHOP targets *BBC3* (PUMA) and *TRIB3* (Supplementary Figure S3B and Supplementary Table S1), which are implicated in stress-induced apoptosis [49–52]. CHOP is a transcriptional target of ATF4, a transcription factor that is translationally upregulated by the PERK-eIF2α arm of the UPR [47]. Consistently, p-eIF2α and ATF4 levels and target gene expression were elevated rapidly upon Combo treatment (Figure 3C, 3D). We also observed increased p-eIF2α and ATF4 levels with Combo treatment when cells were cultured in standard (non-hormone-depleted) FBS (Figure 3E).

Ample evidence in the literature indicates that inhibition of fatty acid synthesis can induce ER stress, particularly if environmental lipids are scarce [42, 43, 53]. Indeed, ACLYi alone induced ATF4 and expression of some ER stress genes; these responses were magnified when ACLYi was combined with ENZ (Figure 3C and 3E, Supplementary Figure S3B, and Supplementary Table S1). Coordination of lipid and protein synthesis is vital for ER homeostasis, and hyper-activation of the PI3K-AKT-mTORC1 pathway drives dependence on *de novo* lipid synthesis for ER homeostasis [42–44, 54, 55]. Notably, a PI3K-AKT signaling pathway gene signature is induced upon Combo treatment (Figure 3B), consistent with prior studies demonstrating that the AR pathway exerts an inhibitory influence over PI3K-AKT pathway [40, 41]. pAKT levels were indeed elevated after Combo treatment (Figure 3F), although signaling downstream of mTORC1 was not similarly elevated (Supplementary Figure S3C), likely due to repression by AMPK (see below). Nevertheless, inhibition of mTORC1 using rapamycin reduced Combo-mediated induction of ATF4 (Figure 3G). Thus, the pro-apoptotic effects of combining ACLY and AR inhibitors could result at least in part from ER stress.

### Concurrent inhibition of ACLY and AR promotes AMPK activation

**Figure 4:**
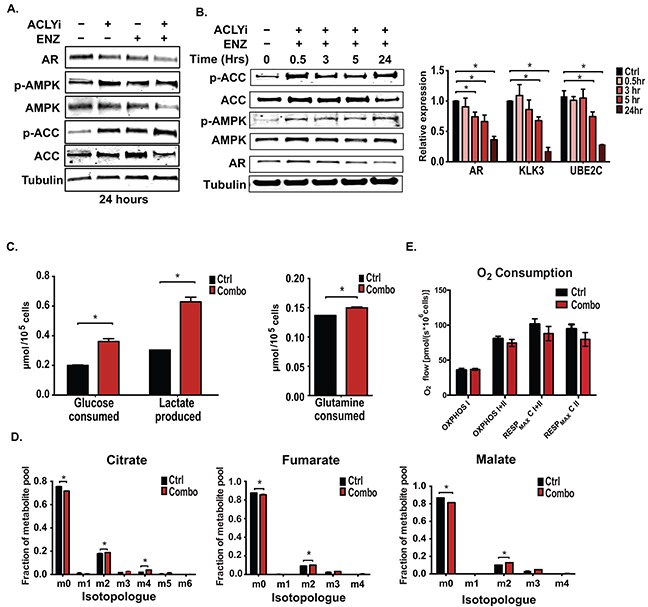
ACLY and AR inhibition promotes AMPK activation without suppressing glucose uptake or oxidation **A.** C4-2 cells were treated as indicated for 24 hours under androgen deprivation and analyzed by Western blot. **B.** C4-2 cells were treated with Combo over a time-course and analyzed by Western blot and Q-RT-PCR. **C.** Consumption of glucose and glutamine from media and production of lactate were analyzed from control and Combo-treated C4-2 cells using a YSI-7100 Bioanalyzer. **D.** [U-^13^C]-glucose labeling of C4-2 cells was conducted, +/− Combo, for 3 hours. Labeling was initiated at the same time as application of the inhibitors. Enrichment in TCA cycle intermediates was assessed. **E.** Oxygen consumption was measured in androgen-depleted C4-2 cells, treated +/− Combo for 24 hours. For all bar graphs, mean +/− SEM of triplicates is graphed; *, p<0.05.

While analyzing the signaling responses to the drug treatments, we noticed that AMPK was potently activated (as assessed by phosphorylation of AMPK and its target ACC1) upon ACLYi or Combo treatment, and that this activation correlated closely with suppression of AR protein levels (Figure 4A and Supplementary Figure S4A). AMPK activation and AR suppression were also observed when shRNA targeting ACLY was used in conjunction with AR antagonism (Supplementary Figure S4B). Recent studies have shown that AMPK activation suppresses AR levels and inhibits prostate cancer cell proliferation and tumor growth [32, 56, 57], and that this response is magnified in the presence of an AR antagonist [32, 57], similar to our observations with ACLY inhibition in this study. Consistently, the AMPK activator AICAR also sensitized C4-2 cells to ENZ (Supplementary Figure S4C).

**Figure 5:**
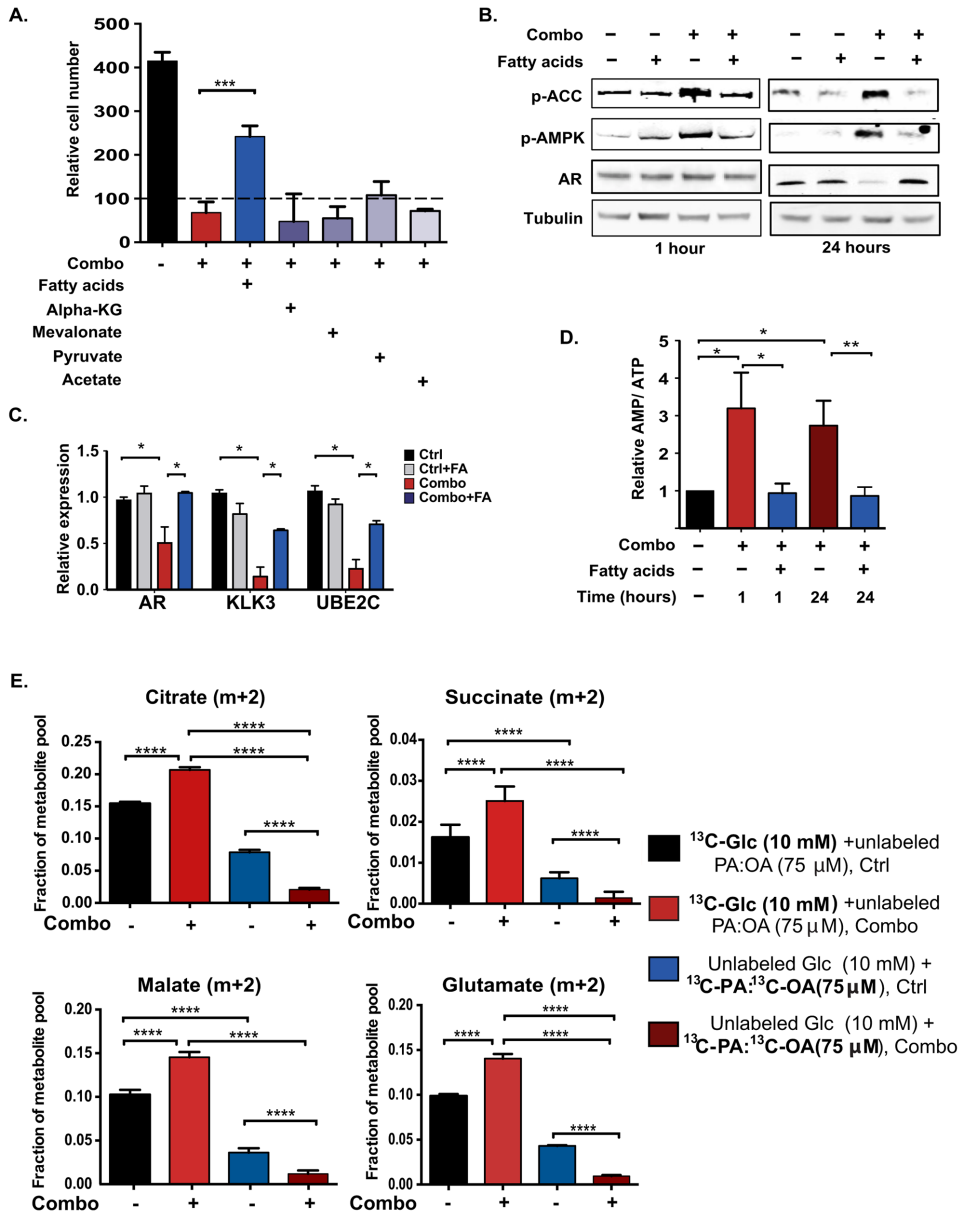
Fatty acid supplementation restores energetic homeostasis in ACLY/AR inhibitor-treated cells, without directly supporting ATP production through β-oxidation **A.** C4-2 cells in androgen-depleted conditions were treated with vehicle, Combo alone, or Combo plus indicated metabolites including fatty acids (75 μM, 50:50 Palmitic acid (PA): Oleic acid (OA), BSA conjugated), dimethyl-α-KG (1 mM), mevalonate (100 μM), Na-pyruvate (5 mM), acetate (500 μM) for 72 hours. After 72 hours, viable cells were counted using trypan blue exclusion and normalized to staring cell number, set to 100. **B.** Immunoblotting was carried out on C4-2 cell lysates treated with vehicle or Combo, +/− fatty acids for 1 hour or 24 hours. **C.** Gene expression in C4-2 cells treated with vehicle or Combo, +/− fatty acids for 24 hours. **D.** C4-2 cells were treated with Combo +/− fatty acids for 1 hour or 24 hours. AMP and ATP were measured by HPLC. Experiment was repeated 4 times, with relative AMP/ATP ratio in untreated cells for each experiment set to 1. 2-tailed paired t-test was conducted. **E.** To assess the relative contribution of glucose and fatty acids to TCA cycle intermediates in control and Combo-treated cells, labeling studies were conducted. Cells were labeled either with 10 mM [U-^13^C]-glucose + 75 μM unlabeled PA: OA or with 75 μM [U-^13^C]-PA: [U-^13^C]-OA + 10 mM unlabeled glucose. Cells were treated +/− Combo concurrent with initiation of labeling, and metabolite enrichment was analyzed after 6 hours. Unless otherwise indicated, mean +/− SEM of triplicates is graphed; *, p<0.05; **, p<0.01, ****, p<0.0001.

We thus investigated the cause of AMPK activation in Combo-treated cells. AMPK activation occurred rapidly upon administration of the Combo, detectable within 30 min (Figure 4B). Although AMPK can be activated in response to diverse stress conditions, the AMP/ATP ratio was also rapidly elevated and sustained following Combo treatment, indicating that energetic stress could be the cause of AMPK activation (Figure 5D). AR protein levels and target gene changes appeared later, beginning to decline about 5 hours following Combo treatment (Figure 4B). Prior studies have demonstrated that AMPK activation inhibits the AR by decreasing its mRNA and protein levels and/or by impairing its nuclear localization [32, 56, 58]. In this context, the effects of the Combo appear to be predominantly on AR levels, since protein levels were drastically reduced but nuclear localization minimally impacted (Supplementary Figure S5A). Together, these data indicate that combined inhibition of ACLY and AR under hormone-depleted conditions rapidly results in energetic stress and AMPK activation, thereby further reducing total AR levels and promoting cell death.

To determine if Combo treatment impacted energy-producing metabolic fluxes, we examined its effects on glucose and glutamine uptake and lactate production. The results showed that Combo treatment produced a marked increase in glucose consumption and lactate production and little change in glutamine consumption (Figure 4C). Glucose oxidation, as assessed by labeling of TCA metabolites from [U-^13^C]-glucose, was only minimally changed in the presence of the Combo (Figure 4D). Moreover, there was no significant change in complex I- or complex I- and II-dependent oxidative phosphorylation, nor in complex II- or complex I- and II-dependent maximal respiration (Figure 4E). Thus, surprisingly, cells experience energetic stress despite having abundant access to and ability to uptake and utilize two major carbon sources, glucose and glutamine.

### Fatty acid supplementation restores energetic homeostasis in cells treated with ACLY and AR inhibitors

To gain insight into the mechanism causing energetic stress, we next attempted to rescue viability and proliferation in the presence of the Combo using supplementation of a panel of metabolites, including fatty acids (50:50 palmitic acid (PA): oleic acid (OA), BSA conjugated), mevalonate, sodium pyruvate, dimethyl α-ketoglutarate, and sodium acetate. Of these metabolites, only fatty acids potently rescued cell number (Figure 5A). BSA alone did not rescue cell number, but both PA and OA individually improved viability (Supplementary Figure S5B). Fatty acid supplementation also reversed the proliferation impairment observed with ACLYi treatment (Supplementary Figure S5C). Exposure to fatty acids prevented Combo-mediated AMPK activation and rescued AR levels in both C4-2 and LNCaP-Abl cells (Figure 5B and Supplementary Figures 5A, 5D). *AR* mRNA and AR target genes were also significantly rescued by fatty acid supplementation (Figure 5C and Supplementary Figure S5E). Additionally, the elevated AMP/ATP ratio was reversed by exogenous fatty acids (Figure 5D).

Prior studies have demonstrated that while fatty acid oxidation (FAO) is conducted minimally in many cancer cells [59], it is important in prostate cancer cells [60–62]. We thus hypothesized that fatty acids rescue viability and suppress AMPK activation in the Combo-treated cells through ATP production via β-oxidation. To test this possibility, we conducted stable isotope labeling experiments using either [U-^13^C]-glucose [with unlabeled 50:50 palmitic acid (PA): oleic acid (OA) present] or a 50:50 mix of [U-^13^C]-PA: [U-^13^C]-OA (with unlabeled glucose present), and examined enrichment in TCA cycle intermediates. After 6 hours of labeling, both glucose and fatty acid carbon were detectable in TCA cycle intermediates in control-treated cells (Figure 5E). In control cells, citrate m+2 (citrate enriched with two carbon-13 atoms) accounted for ~15% of the total citrate pool in the ^13^C-glucose-labeled cells and ~7% of the pool in ^13^C-fatty acid-labeled cells. However, Combo treatment markedly reduced the oxidation of fatty acids in the TCA cycle while increasing the oxidation of glucose (Figure 5E). Thus, FAO does not appear to be central to the mechanism through which fatty acids rescue energetic stress in this context.

### Fatty acids rescue ER homeostasis in combo-treated cells

**Figure 6:**
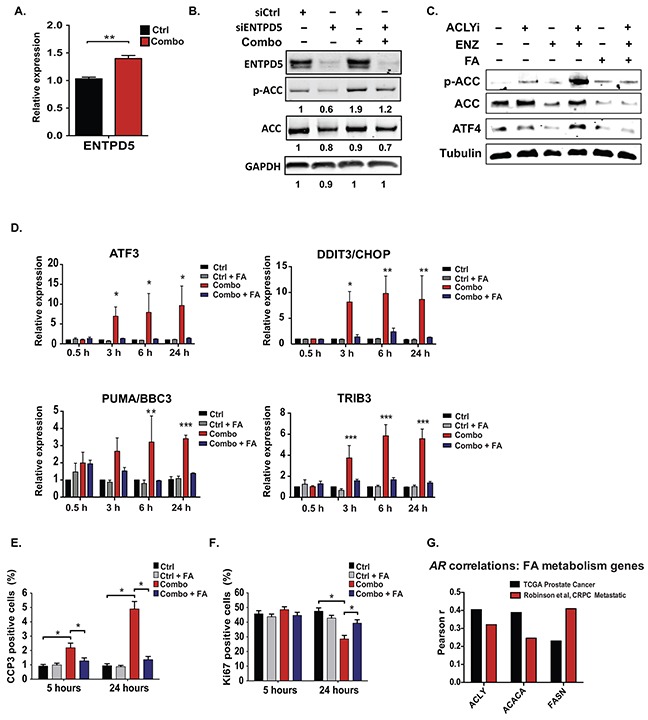
Exogenous fatty acids prevent ER stress and support cell proliferation and viability during treatment with ACLY and AR inhibitors **A.** ENTPD5 gene expression analyzed by Q-RT-PCR after 24 hours Combo treatment in androgen-depleted C4-2 cells. **B.** siRNA targeting ENTPD5 or a non-silencing control siRNA were transfected into C4-2 cells, and after 96 hours, cells were treated +/− Combo for an additional 24 hours before analyzing by Western blot. **C.** LNCaP-Abl cells were treated with indicated inhibitors, +/− 75 μM fatty acids (50:50 PA:OA). **D.** Gene expression was analyzed by Q-RT-PCR at indicated time points, +/− Combo, +/− 75 μM fatty acids (50:50 PA:OA). **E, F.** At indicated time points following Combo treatment, +/− fatty acids, apoptotic marker CCP3 (E) or proliferation marker Ki67 (F) were assessed. For all bar graphs, mean +/− SEM of triplicates is graphed; *, p<0.05; **, p<0.01, ***, p<0.001. **G.** Using the www.cBioportal.org resource [81, 82], AR mRNA levels were tested for correlation with the indicated genes in the prostate cancer TCGA Prostate Adenocarcinoma (mRNA expression, RNA-Seq V2 RSEM) and the Metastatic Prostate Cancer, SU2C/PCF Dream Team (mRNA Expression/polyA, RNA-Seq RPKM) [16, 67]. The key indicates the strength of the correlation, as assessed by Pearson's r. For TCGA data, with n=333, all correlations are significant to p<0.0001. For the metastatic dataset, with n=150, ACLY and FASN correlations are significant to p<0.0001; ACACA correlation is significant to p<0.01.

Since these data suggested that fatty acids do not rescue Combo-treated cells through their direct contribution to mitochondrial ATP production, we next considered the possibility that fatty acids might relieve excess ATP consumption. Protein synthesis and folding and maintenance of ER function are highly energy demanding [63]. As shown in Figure 3, Combo treatment promotes both AKT activation and ER stress. If fatty acids support energetic homeostasis by suppressing ER stress, this could also explain why fatty acids, but not other substrates such as αKG, pyruvate, or acetate, rescued proliferation (Figure 5A). Among genes induced by the drug Combo is ENTPD5 (Figure 6A and Supplementary Table S1), an ER UDPase that activates a cycle of ATP hydrolysis to promote protein glycosylation and folding in AKT-activated cells, in order to promote ER homeostasis [64, 65]. Notably, ENTPD5 is reported to play a major role in ATP consumption and thus, by alleviating allosteric inhibition of PFK1 by ATP, promotes glycolysis [64, 66]. Activation of ENTPD5 is consistent with our findings that the Combo treatment promotes increased glucose consumption and lactate production (Figure 4C), while also increasing the AMP/ATP ratio (Figure 5D). Silencing of ENTPD5 partially suppressed Combo-mediated AMPK activation (Figure 6B), suggesting that ENTPD5 contributes to energetic stress upon Combo treatment, but likely is not the only relevant factor.

We next tested whether fatty acid supplementation could prevent ER stress induction upon Combo treatment. Fatty acid supplementation indeed entirely reversed the effects of Combo treatment on ATF4 levels, as well as ATF4 and CHOP target genes at all time points tested (Figure 6C, 6D and Supplementary Figure S6). Transcriptional targets downstream of IRE1 and ATF6 were also suppressed by fatty acid supplementation (Supplementary Figures S6, S7). Finally, both proliferation and viability were significantly rescued upon fatty acid supplementation (Figure 6E, 6F).

Together these data point to the importance of ACLY function for maintaining ER and energetic homeostasis in CRPC cells. ER homeostasis prevents aberrant AMPK activation, which can suppress the AR [32, 56, 58]. Thus, an ACLY-AMPK-AR feedback loop sustains AR levels in CRPC cells and may contribute to resistance to AR antagonists. If such a feedback loop is relevant in human tumors *in vivo*, a relationship between expression of fatty acid metabolism genes and the AR might be anticipated. To investigate this link, we made use of two recently published human prostate cancer datasets, a prostate adenocarcinoma dataset from TCGA and a metastatic prostate cancer dataset from SU2C/PCF [16, 67]. We found that *AR* mRNA positively correlated with fatty acid synthesis (*ACLY, ACACA, FASN*) genes in both datasets (Figure 6G). Together, the findings of this study suggest that fatty acid metabolism plays a key role in coordinating ER and energetic homeostasis in prostate cancer cells, and that ER stress and AMPK activation caused by concurrent ACLY and AR inhibition leads to enhanced AR suppression, growth inhibition, and cell death.

## DISCUSSION

In this study, we report that inhibiting ACLY sensitizes CRPC cells to AR antagonism. Concurrent inhibition of ACLY and AR results in both inhibition of proliferation and induction of apoptosis, particularly in androgen-depleted conditions. The inhibitor Combo induces energetic stress and AMPK activation, which suppresses AR levels. Thus, this study points to an ACLY-AMPK-AR network that could potentially be targeted to increase the efficacy of current AR-targeted therapies, by impacting metabolic dependencies of prostate cancer cells.

**Figure 7:**
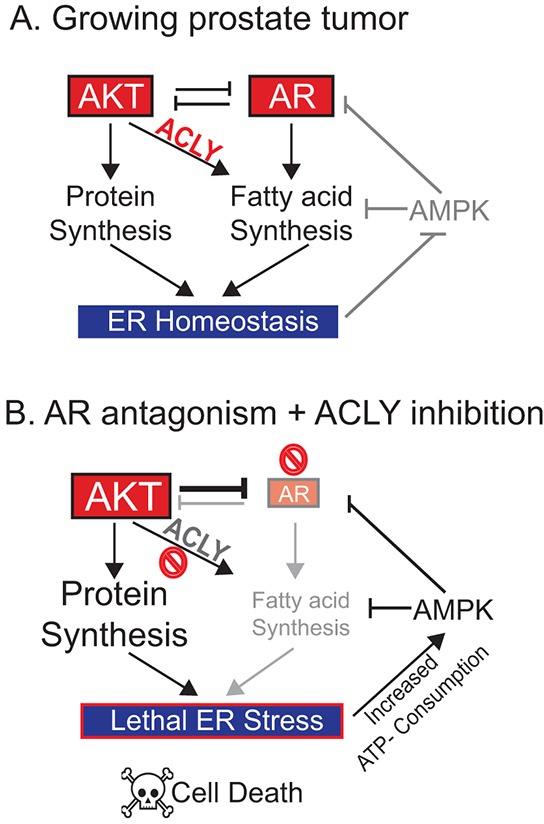
Model: An ACLY-AMPK-AR network sustains ER homeostasis, AR levels, and cell survival in prostate cancer **A.** In proliferating *PTEN* null prostate cancer cells, the demands of oncogenic signaling are met by metabolic rewiring including *de novo* fatty acid synthesis, which supports endoplasmic reticulum (ER) homeostasis and suppresses AMPK activation to promote proliferation. **B.** Upon inhibition of AR, AKT is hyper-activated, placing increased demand on the *de novo* fatty acid synthesis pathways to support ER homeostasis. If ACLY and hence fatty acid synthesis is also inhibited, unresolvable ER stress ensues. In the *PTEN* null context, this can trigger ENTPD5 activation, excessive ATP consumption, and AMPK activation. AR can then be further suppressed by AMPK and AKT, two negative regulators. The combination of AR suppression and pro-apoptotic ER stress may induce cell death. Thus, the ACLY-AMPK-AR feedback loop can be exploited to sensitize CRPC cells to AR antagonism.

The data point to a model in which AR+ prostate cancer cells coordinate cell metabolism and signaling to support the demands of oncogenic signaling (Figure 7A). Upon AR inhibition, AKT is hyper-activated due to alleviation of AR's feedback inhibition on AKT [40, 41]. Elevated AKT activation promotes increased reliance on ACLY-dependent fatty acid synthesis for ER homeostasis [18]; thus, when ACLY is inhibited in this context, an ER stress response is engaged and cannot be resolved. In the context of AKT activation and ER stress, the ER UDPase ENTPD5 is activated to promote protein glycosylation, driving excessive ATP consumption [64, 65]. As the AMP/ATP ratio rises, AMPK is turned on, driving further suppression of the AR. Thus, by sustaining ER homeostasis, fatty acid metabolism plays a crucial role- particularly in *PTEN* null prostate tumors- in maintaining energetic homeostasis and AR levels (Figure 7B). Since 49% of metastatic prostate cancer patients exhibit PI3K/AKT pathway alterations [16], these results are suggestive of possible new therapeutic strategies for these patients, although this requires investigation in vivo.

A prediction of the findings of this study is that compounds targeting other nodes in the ACLY-AMPK-AR network could also be combined with AR inhibition for therapeutic benefit. Consistent with this possibility, both metformin, which activates AMPK and also inhibits fatty acid synthesis in an AMPK-independent manner [68, 69], and a novel AMPK activator (MT 63-78) led to improved anti-tumor effects when combined with AR antagonists [32, 58]. Fatty acid synthesis inhibition warrants further investigation in an *in vivo* context, since tumors frequently outstrip the vasculature and must therefore synthesize fatty acids and other macromolecules to grow in a nutrient-restricted environment [44]. Moreover, ACLY inhibition produces lipid-lowering effects [45, 70, 71], potentially further reducing lipid availability to tumors. Direct AMPK activation is another attractive option, and metformin is appealing in this regard, since it is both safe for patients and the proof-of-concept that metformin and ENZ are effective in suppressing growth of mouse prostate cancer xenograft tumors has been demonstrated [58, 72]. Intriguingly, a novel dual function ACLY inhibitor/AMPK activator has entered clinical trials for metabolic diseases [70]; such a compound could be attractive to test for efficacy in prostate cancer along with an AR antagonist.

Although the dominant effect of ACLY inhibition observed in this study is on fatty acid metabolism, additional effects on acetylation are not ruled out. In particular, restricted histone acetylation has been shown to inhibit the accessibility of chromatin to the AR by attenuating recruitment of the pioneering factor GATA2 [39]. The AR itself is also acetylated, a modification that increases its stability [73]. Since ACLY inhibition on its own has some effect on suppressing protein but not mRNA levels of AR (Figures 2B and 2C), it may be of interest to test a role for ACLY in regulating AR acetylation in the future.

In summary, our data points to the crucial role of ACLY-dependent fatty acid synthesis in coordinating endoplasmic reticulum and energetic homeostasis in prostate cancer cells. By repressing aberrant AMPK activation, fatty acid metabolism thus helps to sustain AR activity, cell survival and proliferation. These studies provide a rationale for further testing of ACLY inhibitors or AMPK activators, particularly in conjunction with AR antagonists, for treatment of CRPC.

## MATERIALS AND METHODS

### Reagents and cell lines

C4-2 cells were a gift of M. Kazanietz, University of Pennsylvania. LNCaP-Abl cells were a gift of Z. Culig, Innsbruck Medical University. Prostate cancer cell lines LNCaP, PC-3, C4-2, and LNCaP-Abl were maintained in RPMI + 10% FBS+ 2 mM L-Gln. 3T3-L1 cells were cultured in DMEM + 10% FBS. All cell lines were confirmed to be mycoplasma-free. Androgen depletion experiments were carried out in phenol-red-free RPMI + 5% charcoal-dextran-treated (CDT) serum + 2 mM L-Gln. For all androgen-deprivation experiments, cells were placed into CDT conditions for 24 hours prior to the addition of treatments. ACLY-inhibitors (BMS-303141 and SB-204990), Bicalutamide, and Enzalutamide (ENZ) were purchased from Tocris (#4609 and 4962), Sigma (#B9061) and Toronto Research Chemicals (#M199800), respectively. ACLY shRNA in pGipZ vector were purchased from Dharmacon (V2LHS_94212, #12; V3LHS_395677, #77). siRNA for ENTPD5 was Dharmacon SMARTpool (L-011235-00-0005).

### Immunoblotting

Cells were lysed in RIPA buffer, in the presence of phosphatase and protease inhibitors and samples were run on NuPage SDS-PAGE gels. The ACLY antibody has been previously described [17]. Commercial antibodies used are listed below.

**Table:** 

Antibody name	Company	Catalogue number
p-AMPK(T-172)	Cell Signaling	2535
Total AMPK	Cell Signaling	2532
Total AKT	Cell Signaling	9272
p-AKT (Thr308)	Cell Signaling	9275
p-AKT (Ser473)	Cell Signaling	4060
p-ACC	Cell Signaling	3661
Total ACC	Cell Signaling	3676
ATF4	Cell Signaling	11815
p-eIF2α	Cell Signaling	3597
AR(N-20)	SantaCruz Biotechnology	sc-816
ENTPD5	Abcam	ab108603

### YSI metabolite measurements

Cells were seeded in CDT-RPMI in 6 well plates with 3×10^5^ cells/well for 24 hours and treated as indicated for an additional 24 hours. Medium was collected at the end of the 24 hours, and glucose, glutamine, and lactate in supernatants were analyzed using a YSI-7100 Bioanalyzer, as previously described [74].

### ^13^C-glucose labeling and GC-MS metabolite measurements

For ^13^C-glucose labeling experiment in Figure 4, 4×10^6^ C4-2 cells were seeded in 10 cm dishes in CDT-RPMI medium overnight. Each culture was then washed with PBS. CDT-RPMI containing 5mM [U-^13^C]-glucose and no un-enriched glucose was added to the cultures for 3 hours either with or without Combo. At the end of the 3-hour period, metabolites were extracted as follows. Acidified cold methanol solution (2.7 ml of methanol, 30μL of 0.1 M HCl and 270μL water) was added with nor-leucine as an internal control. Nor-valine was also added to medium alone for comparison and stored. Cells were collected using a cell scraper and sonicated on a Branson Sonifior 250, at a power setting of 2.5 and a duty cycle of 20% for 30 seconds. The samples were centrifuged at 8,000 rpm at 4°C for 10 min. The metabolite-containing supernatant was evaporated under nitrogen at 40°C. 100 μl of N-tertbutyldimethylsilyl- N-methyltrifluoroacetamide (MTBSTFA) and 100 μl of acetonitrile were added to the dried residue. The samples were heated in 4-ml sealed glass vials at 70°C for 90minutes. The resulting silylated metabolites were transferred to small Eppendorf tubes and centrifuged at 13,000 rpm to remove insoluble materials. One micro-liter of the supernatants was analyzed with an Agilent 7890A/5975A GC-MS system with a DB-5 column. The relative enrichment of metabolites is calculated using IsoCor [75].

### ^13^C-Glucose and ^13^C-Fatty acid labeling comparison and GC-MS metabolite measurements

For ^13^C-glucose and ^13^C-fatty acid labeling experiment in Figure 5, 2×10^6^ C4-2 cells were seeded in 10 cm dishes in CDT-RPMI. On the third day, cells were washed with PBS and plated in medium containing either 10 mM [U-^13^C]-glucose + 75 μM unlabeled palmitic acid and unlabeled oleic acid (both bound to fatty acid free bovine serum albumin (BSA) at a molar ratio of 2:1) or 10 mM unlabeled glucose + 75 μM of both [U-^13^C]-palmitic acid and [U-^13^C]-oleic acid (again BSA bound, from Cambridge Isotopes, Andover, MA). After 6 hours of treatment (control or Combo), metabolites were extracted with 75% cold methanol/water, 2-ml per plate. Cells were collected with a cell scraper and sonicated on a Branson Sonifor 250, at a power setting of 2.5 and 20% duty cycle for 60 seconds. Samples were then centrifuged at 8,500 rpm at 4°C for 15 min. The supernatant was evaporated under nitrogen at 40°C. The dry residue was silylated by adding 50 μL pyridine (anhydrous, 99.8%) and 50 μL N, O-bis(trimethylsilyl)trifluoroacetamide + trimethylchlorosilane (BSTFA + TMCS). Care was taken to exclude water from the mixed samples. They were heated at 55°C for 30 minutes, then centrifuged at 13,000 rpm for 10 minutes. One micro-liter of each supernatant was analyzed with an Agilent 7890A/5975A GC-MS system with a DB-5 column. The relative metabolite enrichment was calculated using IsoCor [75].

### HPLC nucleotide measurements

For analysis of nucleotide alterations, 2×10^6^ cells were seeded in CDT-RPMI in 10cm dishes. Cells were treated with control or Combo +/− fatty acid supplementation (50:50 PA: OA; BSA-conjugated) for desired time (1hr, 24 hrs). Cells were harvested by scraping into cold PBS. Cells were pelleted and lysed using 185 μL cold 0.5M Perchloric acid, then incubated on ice for 2 minutes and spun down to remove protein pellet. Supernatant was collected and neutralized with 42μL 2.5M KOH in 1.5M K_2_HPO_4_, then incubated on ice 2 min and spun again to remove precipitate. Supernatant containing nucleotides was filtered through 0.22uM filter and run on HPLC. Extracts were analyzed by ion pair reverse phase HPLC, as described [76]. Analysis was carried out on a Jasco HPLC system. Nucleotides were detected by UV detector at wavelength 254 nm.

### RNA isolation and Q-PCR analysis

Cells were washed with PBS and RNA isolated using Trizol reagent (Invitrogen), according to manufacturer's instructions. cDNA was prepared and gene expression analyzed as previously described on a ViiA-7 Real-Time PCR system [17]. 18S or B2M were used as internal controls. Primer sequences are listed below:

***KLK3***- Forward: CCCACTGCATCAGGAACAA; Reverse: GCTGTGGCTGACCTGAAATA
***AR***- Forward: CCGGAAATGATGGCAGAGAT; Reverse: CTTCACTGGGTGTGGAAATAGA
***UBE2C***- Forward: TGGTCTGCCCTGTATGATGT; Reverse: AAAAGCTGTGGGGTTTTTCC
***FKBP5***-Forward: CGGTGATTCAGTATGGGA AGATAG; Reverse: GAAAGGCAGCA AGGAGAAATG.
***TMPRSS2***-Forward: TGTGCACCTCAAAGACTA AGAA; Reverse: GCCCATGAACTTC CAGAGTAG.
***E2F1***- Forward: GCTGGACCACCTGATGAATA TC; Reverse: GTCTGCAATGCTACG AAGGT.
***ATF4***- Forward: GGAGATAGGAAGCCAGACTA CA; Reverse: GGCTCATACAGATGCC ACTATC.
***ATF3***- Forward: CTGGAAAGTGTGAATGCTGA AC; Reverse: ATTCTGAGCCCGGACA ATAC.
***DDIT/CHOP***- Forward: CAAGAGGTCCTGTCT TCAGATG; Reverse: GGGTCAAG AGTGGTGAAGATT.
***ERN1***-Forward: CAAGAGGACAGGCTCAAT CAA; Reverse: CCATCATTAGGATC TGGGAGAAAG.
***ATF6***- Forward: GGAGCCACTGAAGGAAGA TAAG; Reverse: GTGCTGCTGGAAGC AATAAAG.
***BBC3/PUMA***- Forward: GTGACCACTGGCAT TCATTTG; Reverse: TCCTCCCT CTTCCGAGATTT.
***XBP1s***- Forward: CCGCAGCAGGTGCAGG; Reverse: GAGTCAATACCGCCAG AATCCA.
***HSPA5/BiP***- Forward: AAGGGGAACGTCTGA TTGG; Reverse: ACGGCAAGAAC TTGATGTCC.
***ERDJ4***- Forward: TCGGCATCAGAGCGCCAA ATCA; Reverse: ACCACTAGTAAAA GCACTGTGTCCAAG.
***ERO1LB***- Forward: TTCTGGATGATTGCTT GTGTGAT; Reverse: GGTCGCTTCA GATTAACCTTGT.
***GADD34***- Forward: CCCAGAAACCCCTACT CATGATC; Reverse: GCCCAGACAG CCAGGAAAT.
***IL8***-Forward: TTTGCCAAGGAGTGCTAAAGA; Reverse: CCACTCTCAATCACTCTCAGTTC.
***TRIB3***- Forward: AGCTGTGTCGCTTTGTCTT; Reverse: CTTGTCCCACAGGGAATCATC.
***B2M***- Forward: ACC TCC ATG ATG CTG CTT AC; Reverse: GGACTG GTCTTTCTATCTCTTGTAC.
***ENTPD5***- Forward: GCCAGGGAAGTGTG TGATAA; Reverse: GCTGTCTGCAA AGCCAAAG
***18S***- Forward: AAATCAGTTATGGTTCCTTTGG TC; Reverse: GCTCTAGAA TTACCAC AGTTATCCAA.

### RNA-Seq bioinformatics analysis

After isolation of RNA using Trizol, Bioanalysis was conducted to confirm quality, using 0.8 RIN value as a cut-off for library preparation. RNA libraries were prepared according to manufacturer instruction using Illumina's TruSeq RNA sample preparation v2 kit using ‘Low Sample’ and the adapters provided in the kit were used.

All the RNA-seq libraries were sequenced by the Functional Genomics Core at the University of Pennsylvania. Sequencing reads were aligned to the UCSC hg19 using RUM pipeline and differential gene expression analysis were performed using edgeR [77, 78]. As an unbiased interrogation of gene regulation, differentially expressed genes (FDR < 0.0001, no fold-change cut off) between any pair of conditions were selected for downstream analysis. Hierarchical clustering was performed based on the average log2-scaled gene expression levels using 1 – “Pearson correlation coefficient” as a distance measure under Ward's linkage criterion. Optimal leaf ordering was also performed for better visualization [79]. After the clustering, four most distinct groups of genes were defined among which two major clusters displayed increasing & gradual response to the combination of ACLYi and ENZ. Gene ontology analysis was performed using Homer and top 10 most significant terms were selected for presentation from each of “Biological Process” and “KEGG Pathway” categories [46]. These data have been deposited in NCBI's Gene Expression Omnibus and are accessible through GSE81796 (http://www.ncbi.nlm.nih.gov/geo/query/acc.cgi?acc=GSE81796).

### Immunofluorescence for AR, Ki67 and CCP3

5×10^4^ cells were seeded in 200 μL CDT-RPMI medium in an 8-well chamber-slide and treated as indicated for 5 hours or 24 hours. After treatment, cells were fixed in 4% paraformaldehyde (PFA), and permeabilized in 0.1% Triton-X100 in PBS for 10 min. After blocking and incubation with primary and secondary antibodies, cells were then incubated with DAPI (1:1000) and mounted. Antibodies used included: Ki67 (1:200; Leica Biosystem), cleaved caspase 3 (1:200; Cell signaling technologies) and 649-conjugated-donkey-anti-rabbit IgG (1:300).

### Oxygen consumption

Oxygen consumption rate was measured using the Oroboros oxygraph O2K system (Oroboros Instruments). Pre-treated cells were trypsinised and rapidly prepared for assay. Briefly, trypsin was stopped by addition of 5% CDT-FCS RPMI and viable cell number determined by Coulter counter (Beckman-Coulter). Cells were centrifuged (300 × g, 5 min, 22°C) and resuspended in PBS. A total of 3×10E6 cells were centrifuged (300 × g, 5 min, 22°C), resuspended in 37°C 0.5 ml MiRO5 mitochondrial respiration medium (Oroboros Instruments,[80]) and injected into the Oroboros measurement chamber containing 1.7 ml of 37°C MiRO5 buffer. For each experiment, control and drug treated cells were measured in parallel. Cells were permeabilized with digitonin (final concentration: 13.6 μg/ml). For complex I oxidative phosphorylation (OXPHOS I), glutamate (9.1 mM), malate (1.8 mM), and ADP (3.6 mM) were added. Complex I-and II-dependent oxidative phosphorylation (OXPHOS I + II) was measured by adding succinate (9.1 mM). Complex I- and II-dependent maximal respiration (RESPmax I + II) was measured by adding oligomycin (2.3 μM) and the uncoupler FCCP (0.9 μM) and complex II-dependent maximal respiration (RESPmax II) was measured after adding rotenone (0.5 μM). Residual oxygen consumption was measured after adding antimycin A (2.3 μM) and subtracted from other measurements. Raw data analysis was performed using Oroboros Dat Lab (5.0).

### Statistical analyses

Student's two-tailed unpaired t-tests were used for all comparisons of 2 samples, unless otherwise indicated. ANOVA was used for comparisons of more than two groups, as indicated in figure legends.

## SUPPLEMENTARY MATERIALS FIGURES AND TABLE




