# Supplementary material for: Targeting ACLY sensitizes castration-resistant prostate cancer cells to AR antagonism by impinging on an ACLY-AMPK-AR feedback mechanism
Source: Oncotarget. 2016 May 27;7(28):43713–30. doi: 10.18632/oncotarget.9666 (PMC5190055; doi:10.18632/oncotarget.9666)
Supplement: Supplementary file 1 [file oncotarget-07-43713-s001.pdf]

Targeting ACLY sensitizes castration-resistant prostate cancer cells to AR antagonism by impinging on an ACLY-AMPK-AR feedback mechanism

SUPPLEMENTARY MATERIALS

REFERENCES

1. Singh D, Febbo PG, Ross K, Jackson DG, Manola J, Ladd C, Tamayo P, Renshaw AA, D'Amico AV, Richie JP, Lander ES, Loda M, Kantoff PW, Golub TR and Sellers WR. Gene expression correlates of clinical prostate cancer behavior. Cancer Cell. 2002; 1:203-209.
2. Cancer Genome Atlas Research Network. Electronic address scmo and Cancer Genome Atlas Research N. The Molecular Taxonomy of Primary Prostate Cancer. Cell. 2015; 163:1011-1025.
3. Gao J, Aksoy BA, Dogrusoz U, Dresdner G, Gross B, Sumer SO, Sun Y, Jacobsen A, Sinha R, Larsson E, Cerami E, Sander C and Schultz N. Integrative analysis of complex cancer genomics and clinical profiles using the cBioPortal. Sci Signal. 2013; 6:pl1.
4. Cerami E, Gao J, Dogrusoz U, Gross BE, Sumer SO, Aksoy BA, Jacobsen A, Byrne CJ, Heuer ML, Larsson E, Antipin Y, Reva B, Goldberg AP, Sander C and Schultz N. The cBio cancer genomics portal: an open platform for exploring multidimensional cancer genomics data. Cancer Discov. 2012; 2:401-404.
5. Lee JV, Carrer A, Shah S, Snyder NW, Wei S, Venneti S, Worth AJ, Yuan ZF, Lim HW, Liu S, Jackson E, Aiello NM, Haas NB, Rebbeck TR, Judkins A, Won KJ, et al. Akt-dependent metabolic reprogramming regulates tumor cell histone acetylation. Cell Metab. 2014; 20:306-319.
6. Decker KF, Zheng D, He Y, Bowman T, Edwards JR and Jia L. Persistent androgen receptor-mediated transcription in castration-resistant prostate cancer under androgen-deprived conditions. Nucleic Acids Res. 2012; 40:10765-10779.

SUPPLEMENTARY FIGURES AND TABLE

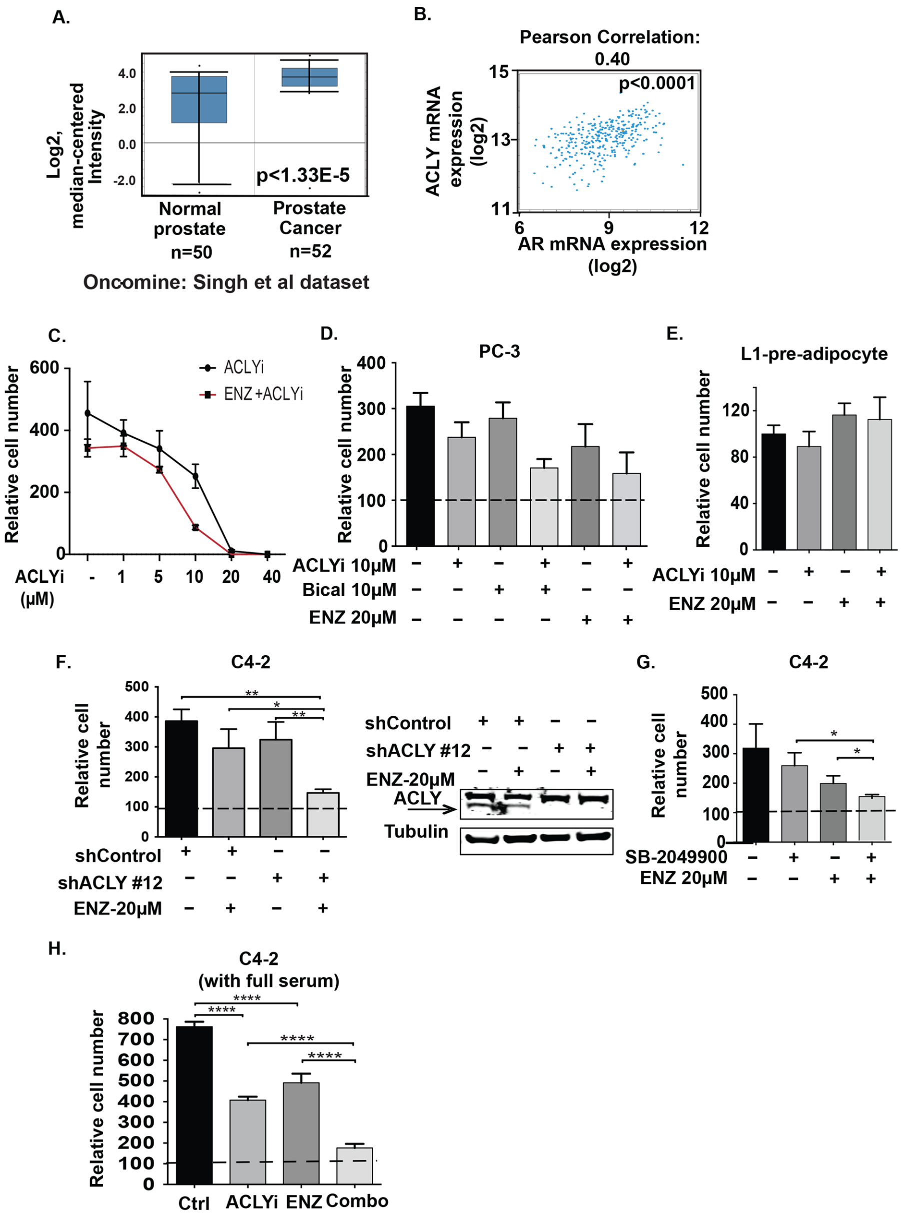

Supplementary Figure S1: ACLY inhibition sensitizes AR+ CRPC cells to AR antagonism. A. ACLY expression in Prostate Carcinoma vs. Normal Prostate Gland in Singh Prostate dataset (accessed using Oncomine) [1]. In this dataset, ACLY expression is higher in Prostate Carcinoma (top 2% of over-expressed genes). **B.** Correlation between AR and ACLY in the TCGA prostate adenocarcinoma dataset, examined using cBioPortal for Cancer Genomics (www.cbioportal.org) [2–4]. **C.** C4-2 cells were treated for 72 hours in indicated doses of ACLYi (BMS-303141), +/- 20 μM ENZ, in androgen-depleted conditions. Viable cells were counted and normalized to starting cell count at time 0, which was set to 100. **D.** PC-3 cells cultured in androgen-depleted conditions were treated as indicated for 72 hours and viable cells counted. **E.** 3T3-L1 pre-adipocytes were treated as indicated for 72 hours and viable cells counted. **F.** C4-2 cells were infected with lentiviral shRNA and treated +/- ENZ for an additional 72 hours in androgen-depleted conditions, and viable cells counted. Panel on the right indicates the silencing efficiency using immunoblotting. **G.** C4-2 cells cultured in androgen-depleted conditions were treated with ENZ, +/- a second ACLYi (25 nM, SB204990), and viable cells counted after 72 hours. **H.** C4-2 cells cultured in RPMI + 5% standard FBS were treated as indicated for 72 hours and viable cells counted. For all bar graphs, mean +/− SEM of triplicates is graphed; *, p<0.05; **, p<0.01; ****, p<0.0001.

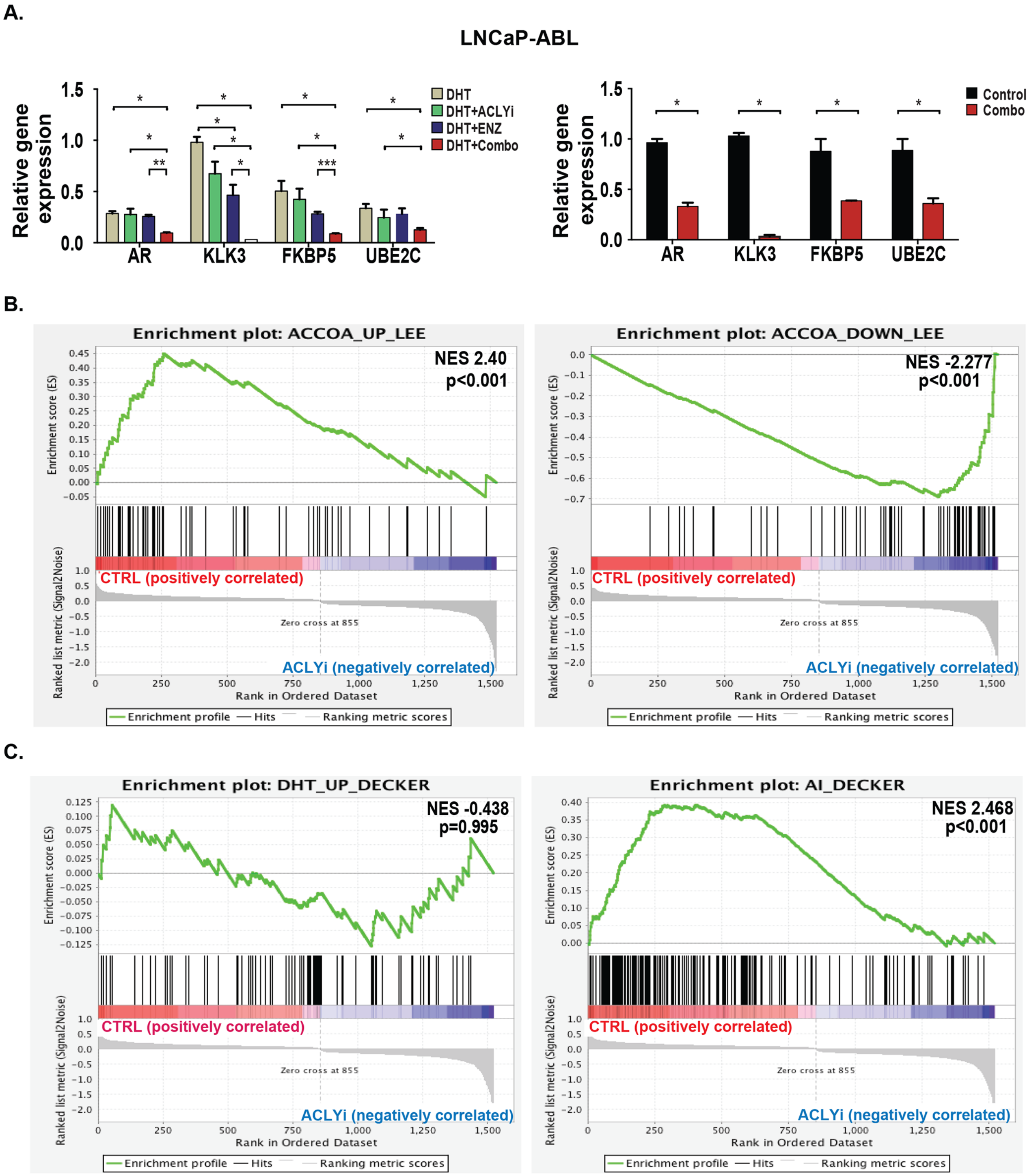

Supplementary Figure S2: ACLY inhibition suppresses "androgen-independent" and "acetyl-CoA-upregulated" genes. A. Androgen-depleted LNCaP-Abl cells were treated with DHT, alone or in combination with ACLYi and/or ENZ, for 24 hours, and gene expression analyzed by Q-PCR (left panel). LNCaP-Abl cells in androgen-depleted conditions were treated +/- Combo for 24 hours. For all bar graphs, mean +/− SEM of triplicates is graphed; *, $p<0.05$; **, $p<0.01$; ***, $p<0.001$. **B.** As assessed by GSEA, ACLYi suppresses "Acetyl-CoA-Upregulated" and enriches for "Acetyl-CoA-Downregulated" genes, as defined by Lee et al [5]. **C.** As assessed by GSEA, ACLYi suppresses "Androgen-independent" but not "DHT-Upregulated" gene signatures, as defined by Decker et al. [6].

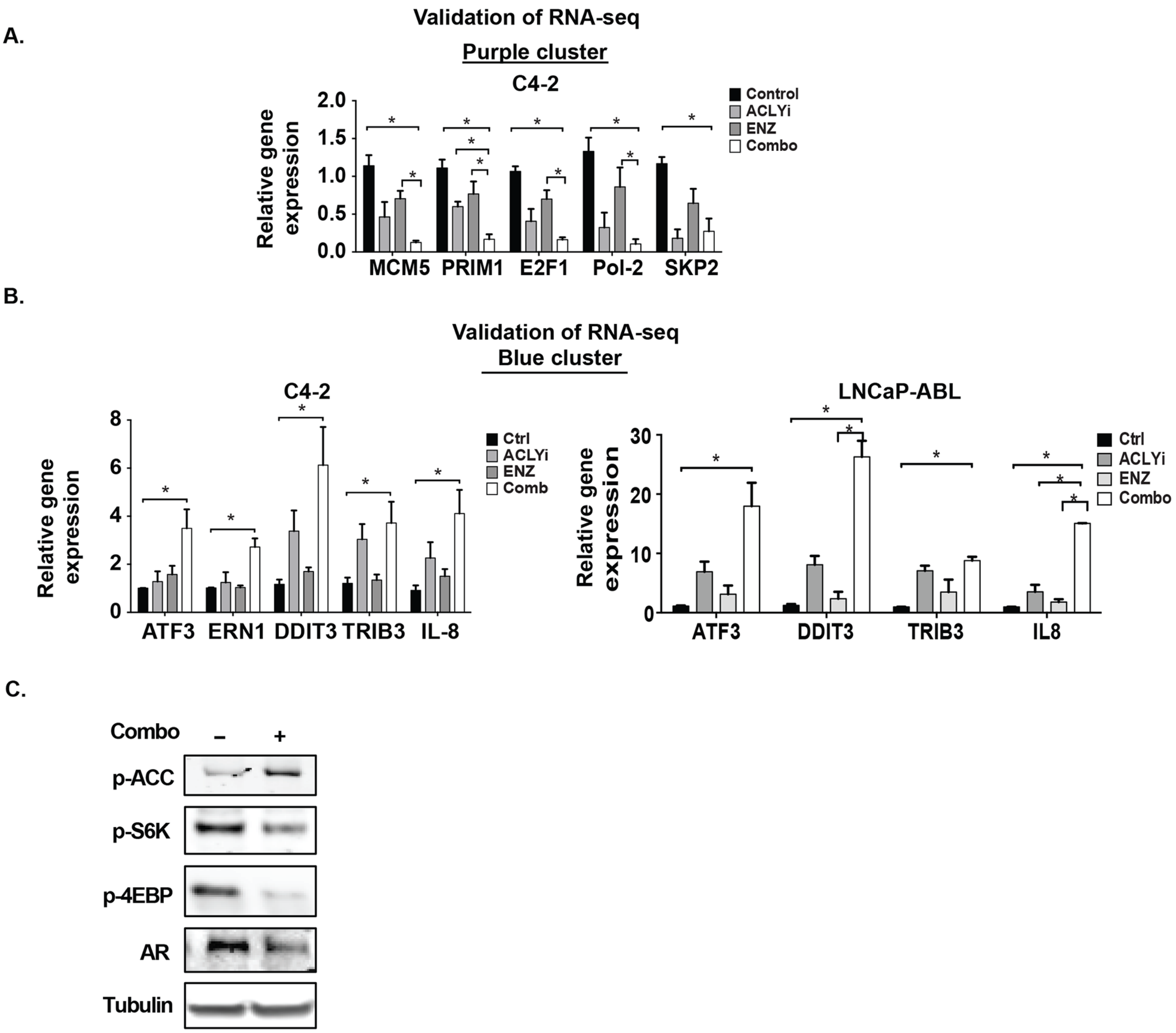

Supplementary Figure S3: Validation of gene expression from RNA-seq. A, B. C4-2 cells treated as in the RNA-Seq experiment were used for Q-PCR analysis of gene expression in both the purple cluster (A) and the blue cluster (B, left panel). Changes in genes in blue cluster were also confirmed in LNCAP-ABL cells (B, right panel). **C.** C4-2 cells were treated for 24 hours with Combo in androgen-depleted conditions and analyzed by Western blot.

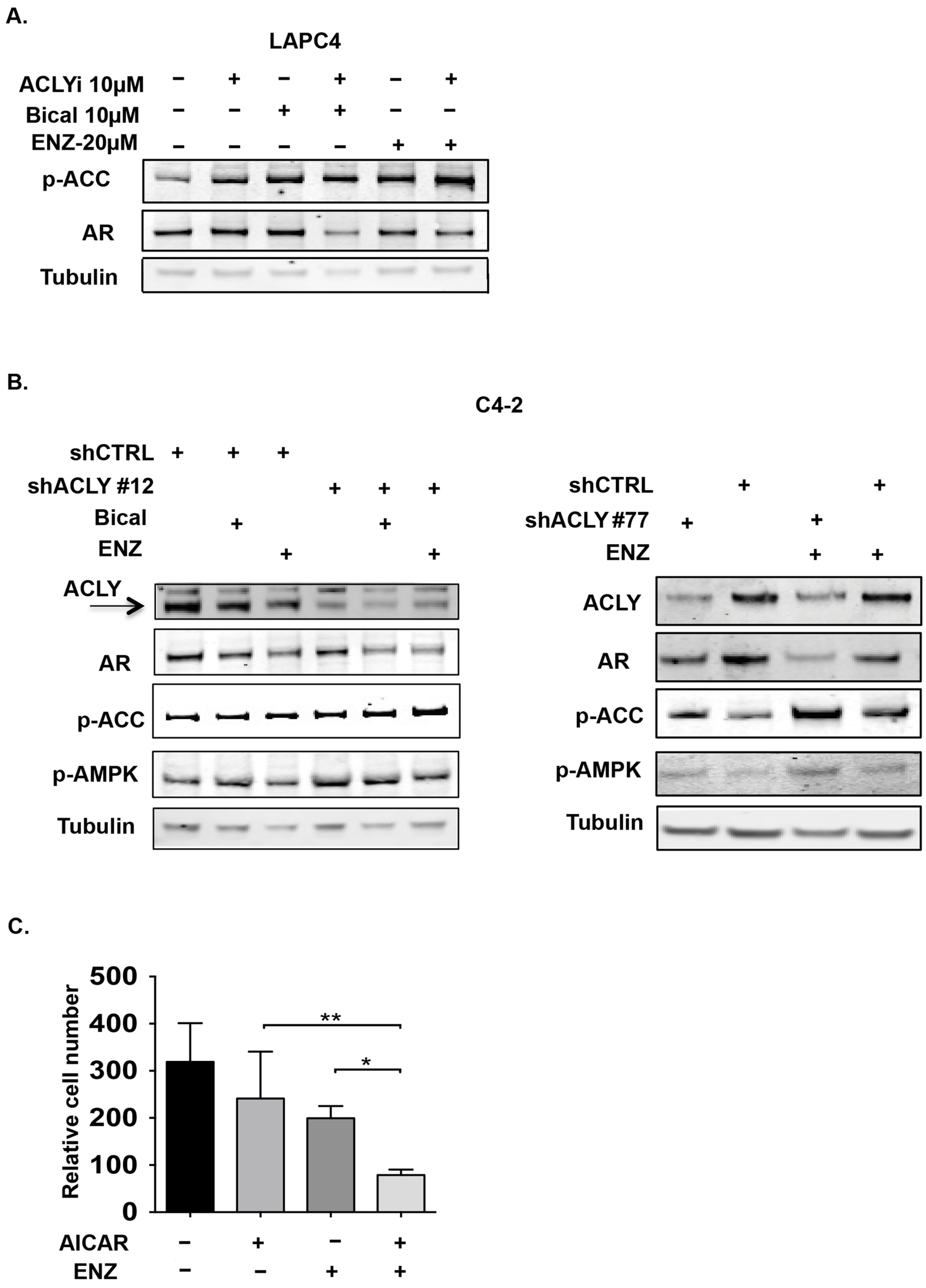

Supplementary Figure S4: Concurrent inhibition of ACLY and AR induces energetic stress and further suppression of AR protein in prostate cancer cells. A. LAPC4- cells were treated with ACLYi in combination with either of the AR-inhibitors (Bicalutamide, 10µM or Enzalutamide, 20µM) under androgen depleted conditions and analyzed by Western blot. **B.** C4-2 cells were infected with lentiviral shACLY #12 for 2 days and then treated with Bical (10µM) or ENZ (20µM) in androgen-depleted conditions (left panel). Results were validated with a second shRNA (shACLY #77) (right panel). Signaling was assessed by Western blot. **C.** Androgen-depleted C4-2 cells were treated for 72 hours +/- AICAR, +/- ENZ and viable cells counted.

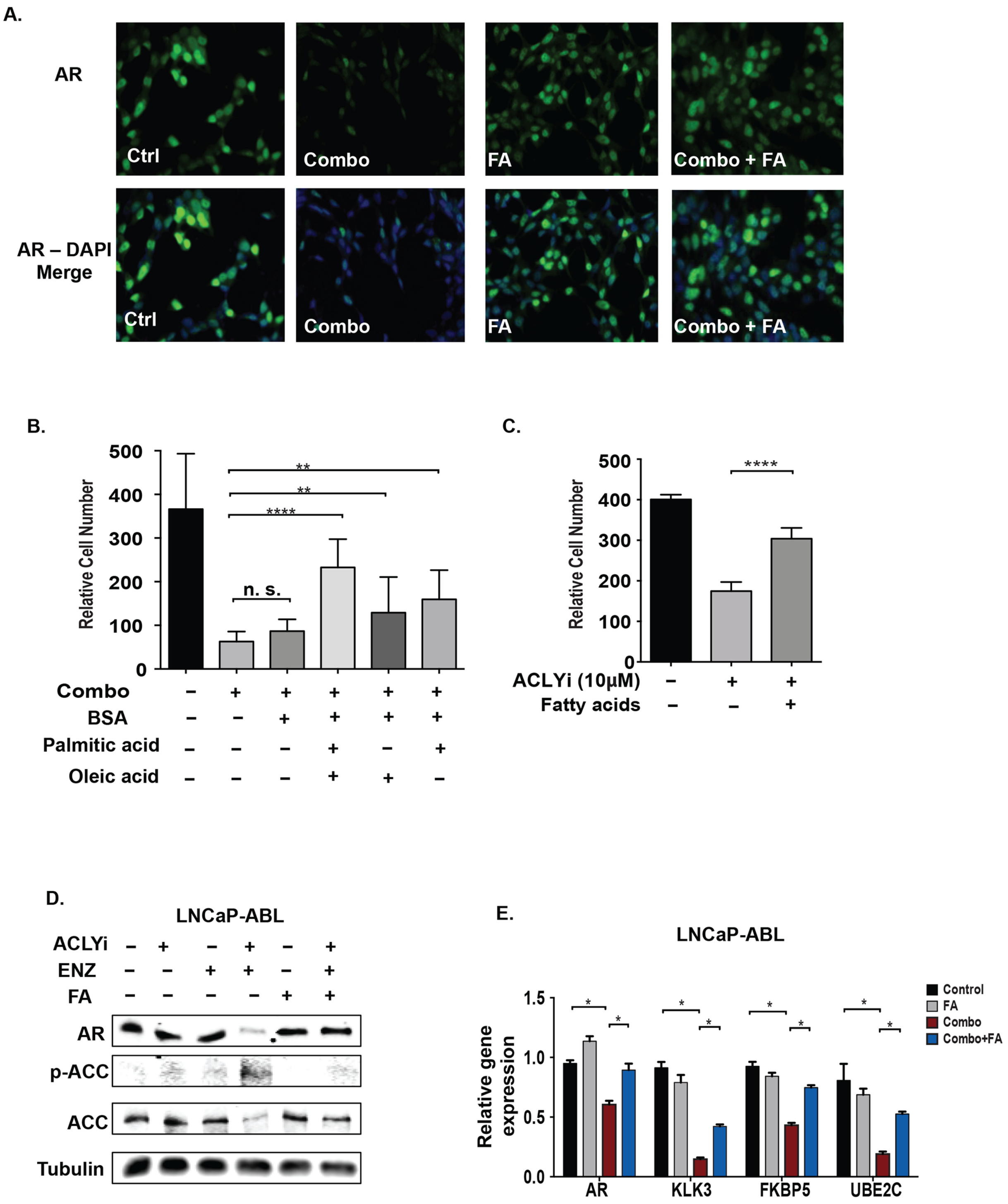

Supplementary Figure S5: Fatty acid supplementation restores AR activity in the presence of ACLY and AR inhibitors. **A.** Immunofluorescent imaging of AR protein levels was conducted in C4-2 cells in CDT conditions 24 hours after Combo treatment, +/- fatty acids (50:50 PA: OA, BSA conjugated). **B, C.** Androgen-depleted C4-2 cells were cultured for 72 hours in androgen depleted conditions in indicated conditions and viable cells counted. PA and OA were BSA-conjugated. **D.** CRPC cell line LNCaP-Abl was treated as indicated for 24 hours in androgen-depleted conditions and analyzed by Western blot. **E.** LNCaP-Abl cells were treated as in (D) and gene expression analyzed by Q-PCR.

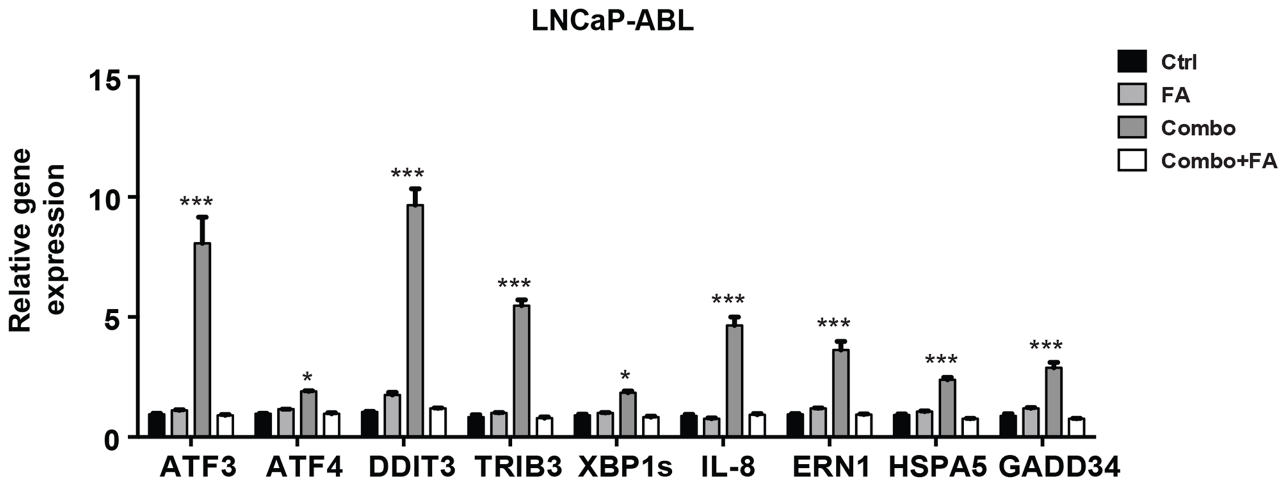

Supplementary Figure S6: Fatty acids rescue ER stress induction in LNCaP-Abl CRPC cells. Gene expression analyzed by Q-PCR in LNCaP-Abl cells treated with Combo, +/− fatty acid supplementation for 24 hours. Mean +/− SEM of triplicates is graphed, and ANOVA was used to compare Combo to the rest of the samples, *, $p<0.05$; ***, $p<0.001$.

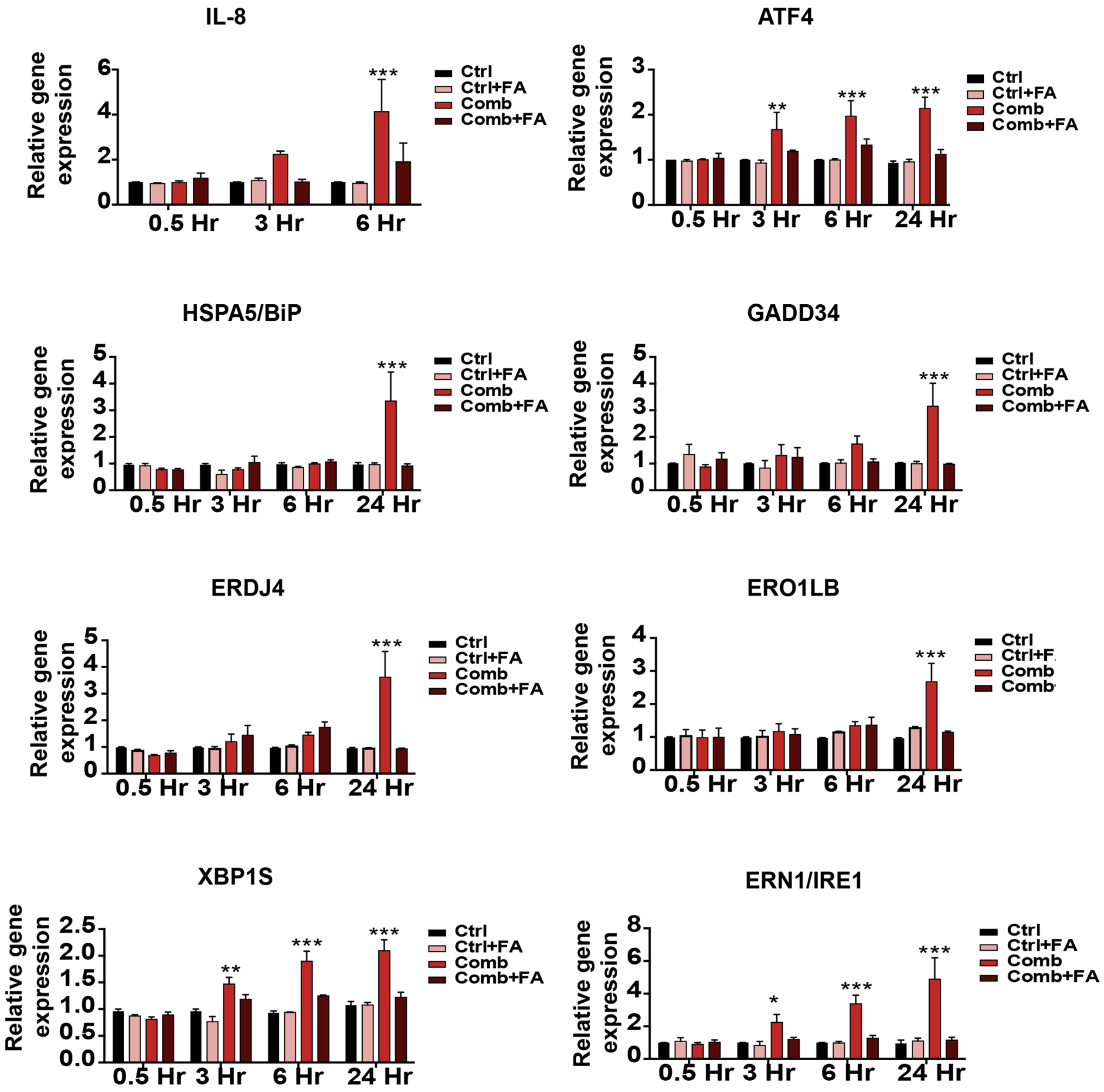

Supplementary Figure S7: Fatty acids rescue ER stress induction in C4-2 cells, additional genes. C4-2 cells were treated with Combo, +/− fatty acid supplementation concurrently for 0.5, 3, 6, and 24 hours and gene expression analyzed by QPCR. Mean +/- SEM of triplicates is graphed, and ANOVA was used to compare Combo to the rest of the samples, *, $p<0.05$; ***, $p<0.001$.

Supplementary Table S1: Datafile for RNA-Seq significantly regulated genes and their cluster IDs.

See Supplementary File 1
